# Editorial: Highlights in assisted reproduction 2023/24

**DOI:** 10.3389/frph.2025.1648172

**Published:** 2025-08-01

**Authors:** Eitan Lunenfeld, Johnny S. Younis

**Affiliations:** ^1^ Adelson School of Medicine, Ariel University, Ariel, Israel; ^2^ Tzafon Medical Center, Poriya, and the Azrieli Faculty of Medicine, Bar-Ilan University, Safed, Israel

**Keywords:** assisted reproduction (ART), GnRH analogues or agonists, IVF, recurrent implantation failures (RIF), ethical slippery slope

**Editorial on the Research Topic**
Highlights in assisted reproduction 2023/24

Since the introduction of Assisted reproduction first by ovulation induction and intra- uterine insemination and then by *in vitro* fertilization, many changes have occurred with the aim of increasing efficacy and safety. One of the highlights of ovarian stimulation protocols was the introduction of GnRH analogues into the stimulation protocols to prevent “premature LH surges”, which at that time caused around 30% ovum pick up cancellation rates. The GnRH agonists were the first to evolve but the protocols were long and cumbersome and had a relatively high rate of ovarian hyperstimulation syndrome (OHSS). Later on, when the GnRH antagonists were introduced, non-inferiority efficacy was demonstrated compared to the agonists. Furthermore, the antagonists reduced the OHSS rates both by reducing the need for high gonadotropin dosage and by providing the option to trigger final oocyte maturation using GnRH agonist. The study by Xinyue et al. compares in a retrospective cohort study, pregnancy and OHSS rates between two different GnRH antagonists’ products Cetrorelix and Ganirelix in IVF/ICSI antagonist “flexible protocols”. Both Cetrorelix and Ganirelix demonstrate comparable live birth rates. However, in this retrospective study, Ganirelix showed a higher overall OHSS incidence (1.1% vs. 0.4%, *P* = 0.01). Optimization and personalizing ART treatments is another challenge. Blockeel et al. tried in their AMPLITUDE Delphi consensus paper to optimize treatments in assisted reproduction technology. A Scientific Committee developed eleven statements for patient profiles corresponding to predicted ovarian responses. This was distributed among French and Belgian fertility specialists (52 responders). Consensus was reached when ≥66.7% of participants agreed or disagreed. A consensus agreement was reached for personalizing the initial dose of gonadotropin, taking age, weight, body mass index, nature of the cycle, and the decision to perform a fresh transfer or a freeze-all strategy into consideration. The respondents preferred a fresh transfer for low and normal responders and a freeze-all strategy in case of high risk of hyperstimulation, newly diagnosed uterine or tubal pathology, and premature progesterone elevation. A consensus was reached for 10–15 oocytes as the optimal oocyte target from the first round of voting. A consensus was also reached to increase the gonadotropin dose in case of insufficient response, and preferred a GnRH antagonist protocol for a subsequent cycle in case of excessive response. Finally, a consensual answer was obtained for using LH/hCG activity in case of hypogonadotropic hypogonadism, advanced age, inadequate response during first stimulation, and suspected FSH receptor polymorphism.

Another challenge in Assisted Reproduction is increasing the live birth rate on the one hand and exploring the mechanism and management protocols in cases of recurrent implantation failures (RIF). There is no question that Uterine Natural Killer cells (uNK) play a role in physiological trophoblast invasion and angiogenesis; however their role in RIF is not substantial. When endometrial scratching was first introduced to increase implantation rates, especially in cases with RIF it showed encouraging results, although the biological mechanism was not very clear. Furthermore, big RCTs trying to modulate the intrauterine immune response by IV intralipid and increasing live birth rates are lacking. Mrosk et al., in their retrospective study, did not show convincing evidence for the “add on” endometrial scratching on live birth rates. In this study, patients previously identified with increased uNK cells (>300 uNK cells/mm^2^) were offered an off-label intralipid infusion therapy that did not demonstrate any benefit.

When IVF was first introduced by Edward and Steptoe, it first used natural cycles and Laparoscopic oocyte retrieval to treat tubal disease. With the development of ART sophisticated ovarian stimulation protocols were developed as well as trans vaginal ultrasound guided oocyte retrieval. Furthermore, the indications for IVF were extended to male infertility as well as oocyte cryopreservation for the purpose of fertility preservation and surrogacy. The case report by Ingold et al. demonstrates the feasibility and safety of combined laparoscopic and transvaginal oocyte retrieval in a woman with vaginal recurrence of cervical adenocarcinoma following radical hysterectomy, using “random start” ovarian stimulation protocol employing Aromatase Inhibitors, Gonadotropins, GnRH antagonist and GnRH agonist for final oocyte maturation to eliminate OHSS. This case report represents a “tailored made” treatment management for a specific, unusual case.

As assisted reproduction continues to evolve, future studies should focus on refining personalized treatment strategies, improving safety profiles of stimulation protocols, and identifying evidence-based interventions for challenging cases such as recurrent implantation failure. High-quality, prospective research is essential to validate current practices, clarify immunological contributions, and expand access to individualized care across diverse patient populations.

There is no question that we have not yet reached the goal of efficacy, and more challenges are ahead. However, it is crucial that while trying to conquer the challenges, we do not slip into the ethical slippery slope and always remember that we are dealing with germ lines, which have consequences for the next generation.

